# Au-PDA@SiO_2_ core-shell nanospheres decorated rGO modified electrode for electrochemical sensing of cefotaxime

**DOI:** 10.1038/s41598-019-55517-9

**Published:** 2019-12-13

**Authors:** M. Z. H. Khan, M. Daizy, C. Tarafder, X. Liu

**Affiliations:** 1 Department of Chemical Engineering, Jashore University of Science and Technology, Jashore, 7408 Bangladesh; 2 College of Chemistry and Chemical Engineering, Henan University, Kaifeng, 475004 China

**Keywords:** Materials science, Nanoscience and technology

## Abstract

In this work, we have successfully synthesized core-shell structured Au-PDA@SiO_2_ nanospheres and decorated on reduced graphene oxide (rGO) modified glassy carbon electrode for the electrochemical detection of cefotaxime. The one-pot hydrothermal method was used to synthesis core-shell nanostructures by loading Au nanoparticles on polydopamine (PDA) coated SiO_2_ nanospheres. The as-prepared Au-PDA@SiO_2_ nanospheres were used to fabricate electrochemically reduced graphene oxide (rGO) modified glassy carbon electrode (Au-PDA@SiO_2_/rGO/GCE) for electrochemical determination of cefotaxime. Scanning electron microscopy, powder x-ray diffraction, transmission electron microscopy, and Fourier-transform infrared spectroscopy were used to confirm the structure and morphology of the as-prepared nanospheres. Cyclic voltammetry (CV) and electrochemical impedance spectroscopy (EIS) were performed for electrochemical characterizations different modified electrodes. It was revealed that the nanocomposite modified electrodes exhibited excellent electrochemical performances for electrooxidation of target analytes and could achieve ultra-sensitive detections. A linear relationship was observed between peak currents and concentrations in the ranges of 1.0 × 10^−9^ to 5.0 × 10^−8^ M (*R*^2^ = 0.9877), and 1.0 × 10^−7^ to 5.0 × 10^−6^ M (*R*^2^ = 0.9821) for cefotaxime with a detection limit (S/N = 3) of 1.0 × 10^−10^ M. It can be deduced that the proposed sensor is suitable for the sensitive detection of cefotaxime in pharmaceutical samples.

## Introduction

Cefotaxime (CEF) belongs to the class of beta-lactam third generation cephalosporin antibiotic and own a broad spectrum of activity against both Gram positive and negative bacteria^1^. This semisynthetic antibiotic widely used to treat urinary tract infections, gonorrhea, pneumonia and pelvic inflammatory diseases^2^. Because of its crucial role in numerous pharmaceutical and pathological processes, it is essential to develop selective and sensitive detection and quantification of CEF. To date, several analytical methods have been reported for detection of CEF including chromatography^3^, spectrophotometry^4^, fluorescence^5^, electrochemistry^1,2^ etc. However, most of the analytical methods are costly, time consuming and need tedious pretreatment. On the other hand, electrochemical detection has been found more attractive due to their simplicity, higher sensitivity, low cost and selectivity over conventional methods.

Recently, graphene and reduced graphene oxide have attracted significant interest worldwide due to its extraordinary electrochemical properties, high surface area and high charge-carrier mobility^6–8^. Reduced graphene oxide was reported to provide an effective sensing platform for selective detection of bio-entities due to their tunable functionalities on their basal planes^9–12^. On the other hand, Silica nanoparticles based materials with tunable pore structures attracted significant attention in various electrochemical sensors^13^. Several works have been reported on polymer coated SiO_2_ nano-spheres^14–17^. Among them, dopamine has attracted considerable attention due to its spontaneous oxidative polymerization to form polydopamine (PDA)^18–20^. PDA has been exploited as an adhesion layer to immobilize self-assembled monolayer, metal films^21^, biological molecules^22^, etc. to form biosensors^23–27^. Moreover, gold nanoparticles (AuNPs) have high surface reaction activity, strong adsorption ability, and good electrical properties. The incorporation of AuNPs was reported to enhance the sensitivity and selectivity of modified electrode platform^28^.

In this work, we present a simple one-step controllable synthesis of core-shell structured Au-PDA@SiO_2_ nanospheres and decorated on rGO modified glassy carbon electrode for the electrochemical detection of cefotaxime. The morphology and electrochemical properties of the prepared nano-sphere was investigated. Moreover, the real sample was analyzed to study the potential application of the modified electrode.

## Materials and Method

### Materials

Tetraethylorthosilicate (TEOS), tetrachloroaurate (HAuCl_4_), trihydrate ethanol and ammonium hydroxide (NH_3_.H_2_O, 25–28%) were purchased from Aladdin Reagent, Shanghai. Modified Hummers’ method was used to produce graphene nanosheet from natural graphite powder and described in our previous paper^8^. Other chemicals were of analytical grade and used without further purification.

### Preparation on AuNPs colloidal solution

Typically, 212 ml of DI water was vigorously stirred under reflux and 25 ml of 2.54 mM HAuCl_4_ solution was added. The resultant solution was stirred until the boiling point achieved. Then, 12.5 ml of 10 mg/ml^−1^ sodium citrate solution was added and the system was refluxed for 30 min, cooled at room temperature and kept in refrigerator at 4 °C.

### Synthesis of Au-polydopamine@SiO_2_ core-shell nanospheres

StÖber method was used to synthesis SiO_2_ particles using tetraethylorthosilicate (10.8 ml) and ammonium hydroxide (17.0 ml). Later, the as-synthesized SiO_2_ was dispersed in the buffer (Tris; 8.5 pH at 25 °C) and sonicated for 30 min. Then, 2 mg/ml dopamine hydrochloride was added in the solution with continuous stirring at 250 rpm for 6 h. Finally, pre-prepared AuNPs solution (0.5 mM) was added in the mixture during stirring and continued for 4 h. After completion of reaction, the mixture was centrifuged and washed several times to remove unattached AuNPs from PDA@SiO_2_ surface.

### *Preparation of* Au-PDA@SiO_2_/rGO/GCE *electrode*

Before modification, the bare GCE electrode was polished with 0.05 μ m alumina slurry. Later, the polished electrode was washed stepwise in ultrasonic bath with nitric acid (1:1), ethanol, and deionized water respectively. Finally, the cleaned electrode was rinsed with ultra-pure water and dried on air. The rGO was electrodeposited on GCE electrode through cyclic voltammetries between −1.3 and 0.7 V vs. Ag/AgCl for 15 cycles with a scan rate of 50 mVs^−1^. After electrodeposition of rGO on GCE, the rGO/GCE electrode was washed with ultrapure water and air dried. On the other hand, 5 mg of Au-PDA@SiO_2_ nanocomposite was dispersed in 1 ml of ethanol and dropwise cast (20 µl) on the pretreated rGO/GCE electrode surface. The modified electrode was named as Au-PDA@SiO_2_/rGO/GCE modified electrode and used for electrochemical studies.

### Apparatus

Corrtest electrochemical workstation (CS-300, Wuhan, China) was used for all electrochemical measurements. Surface morphology measurements were done using scanning electron microscopy (SEM) (Hitachi S-3000H, Japan), transmission electron microscope (TEM) (JEOL JEM-2100 model), and powder X-ray diffraction (XRD) (Bruker D8 Advance, Germany). Nitrogen (N_2_) atmosphere and 25 °C temperature were maintained during electrochemical measurements.

## Results and Discussion

### Characterization of Au-polydopamine@SiO_2_ core-shell nanospheres

**Figure 1:**
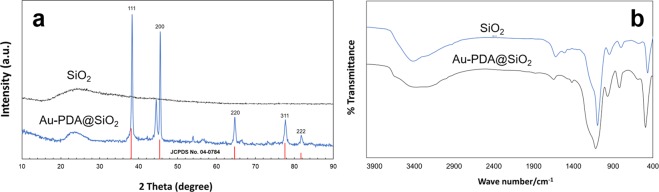
XRD pattern (**a**) and FTIR spectra (**b**) of SiO_2_ and Au-PDA@SiO_2_ nanocomposite.

The wide-angle XRD patterns give the chemical composition and the crystalline nature of the as-prepared nanoparticles as illustrated in Fig. 1a. A broad scattering maximum centered at 22.5° corresponding to amorphous silica as reported in previous literature^29^. The decreased intensity of SiO_2_ nanoparticles confirms the PDA coating. From the XRD pattern of Au-polydopamine@SiO_2_, the peaks at 2*θ* = 38.07°, 44.24°, 64.43°, and 77.35° were indexed to (111), (200), (220), and (311) sets of planes of the face centered cubic structure of AuNPs (with reference to JCPDS File no. 04–0784) as shown in Fig. 1a.

Figure 1b shows the FTIR spectra of SiO_2_ and Au-PDA@SiO_2_ nanosphere. For SiO_2_, the broad peak observed at 3415 cm^−1^ is assigned to O-H stretching in silica, whereas the peak at 1619 cm^−1^ is for O-H scissor bending vibration. On the other hand, the sharp peaks observed at 1109 and 799 cm^−1^, respectively can be assigned to O-H ions as reported in literature^30^. After polydopamine coating on silica nanoparticle, indole aromatic ring vibrations were noticed at 1628 cm^−1^ as reported by earlier researchers^28,31^. Moreover, the presence of PDA was confirmed by the peak at 3407 cm^−1^.

**Figure 2:**
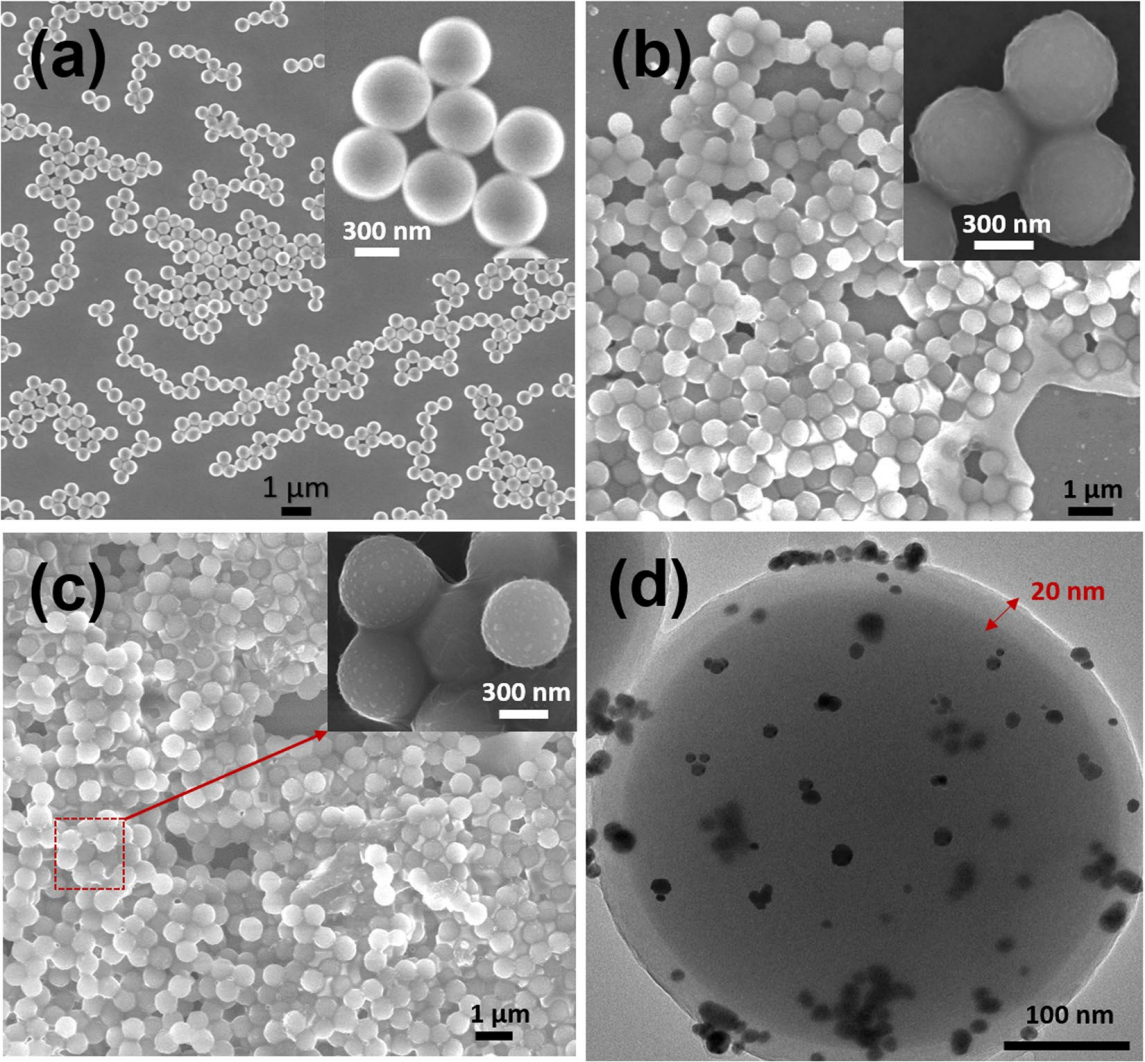
SEM images of (**a**) freshly prepared SiO_2_ nanoparticles; (**b**) PDA@SiO_2_ after coating with polydopamine; (**c**) AuNPs decorated on PDA@SiO_2_ nanoparticles. The insets show the high magnification images. The high magnification TEM image of Au-polydopamine@SiO_2_ core-shell nanospheres (**d**) clearly indicates the 20 nm layer of PDA coating and AuNPs decoration on SiO_2_ particle.

SEM and TEM measurements were carried out to confirm the structural morphology of as-prepared nanocomposites. Figure 2a shows the SEM image of SiO_2_. A well-defined spherical morphology was observed for SiO_2_ particles with an average particle size of 400 nm. After the polymerization of dopamine on SiO_2_, a thin layer appears on the surface of the SiO_2_. It is difficult to distinguish the thin thickness shell of PDA due to lower contrast comparing with SiO_2_. After polydopamine coating of SiO_2_ particles, the PDA@SiO_2_ has a size of 450 nm as shown in Fig. 2b. The AuNPs decorated on PDA@SiO_2_ nanoparticles is shown in Fig. 2c. The presence of AuNPs on the surface of PDA@SiO_2_ was confirmed by the clear bright spots from TEM images. Figure 2d also confirms the thickness of PDA shell (about 20 nm) on the surface of SiO_2_ shell.

### Electrochemical characterization of the modified electrode

**Figure 3:**
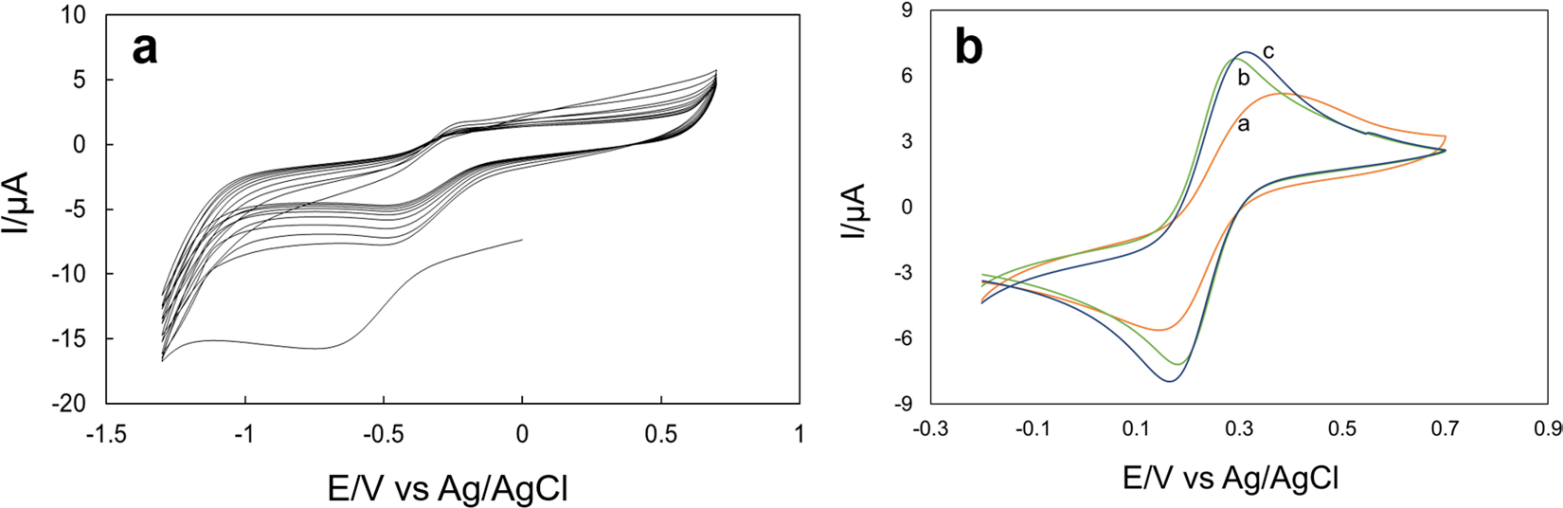
Cyclic voltammograms for (**a**) the electrochemical deposition/reduction of 0.5 mg ml^−1^ GO nanosheets in 0.1 M PBS solution (pH 7.0) on GCE electrode at the scan rate of 50 mV/s for 15 cycles; (**b**) the bare and modified electrodes measured in 0.1 M L^−1^ KCl including 5.0 mM L^−1^ Fe(CN)_6_^3−/4−^: (**a**) bare GCE, (**b**) rGO/GCE, and (**c**) Au-PDA@SiO_2_/rGO/GCE.

Figure 3a represents CVs of the rGO electrodeposition on the GCE electrode (−1.3 and 0.7 V vs. Ag/AgCl; scan rate of 50 mVs^−1^). The electrochemical performance of the bare and different modified electrodes was tested via CV in 5.0 mM L^−1^ Fe(CN)_6_^3−/4−^ electrolytes as shown in Fig. 3b. Well defined redox peaks were observed for both bare and modified electrodes corresponds to Fe(CN)_6_^3−/4−^. A peak to peak separation (*ΔE*_*p*_) value of 211 mV was calculated for bare electrode that indicates slow electron-transfer kinetics at the surface. However, after rGO modification, the *ΔE*_*p*_ values were 85 mV suggesting larger electroactive surface. While after Au-PDA@SiO_2_ deposition (Au-PDA@SiO_2_/rGO/GCE), the *ΔE*_*p*_ value was 77 mV, which is larger than rGO/GCE but much higher than bare electrode. It can be deduced that due to synergetic amplification, conductive graphene sheets conjugated Au-PDA@SiO_2_ nanosphere effectively facilitate electron transfer rate.

### Electrochemical behavior of CEF on modified electrodes

**Figure 4:**
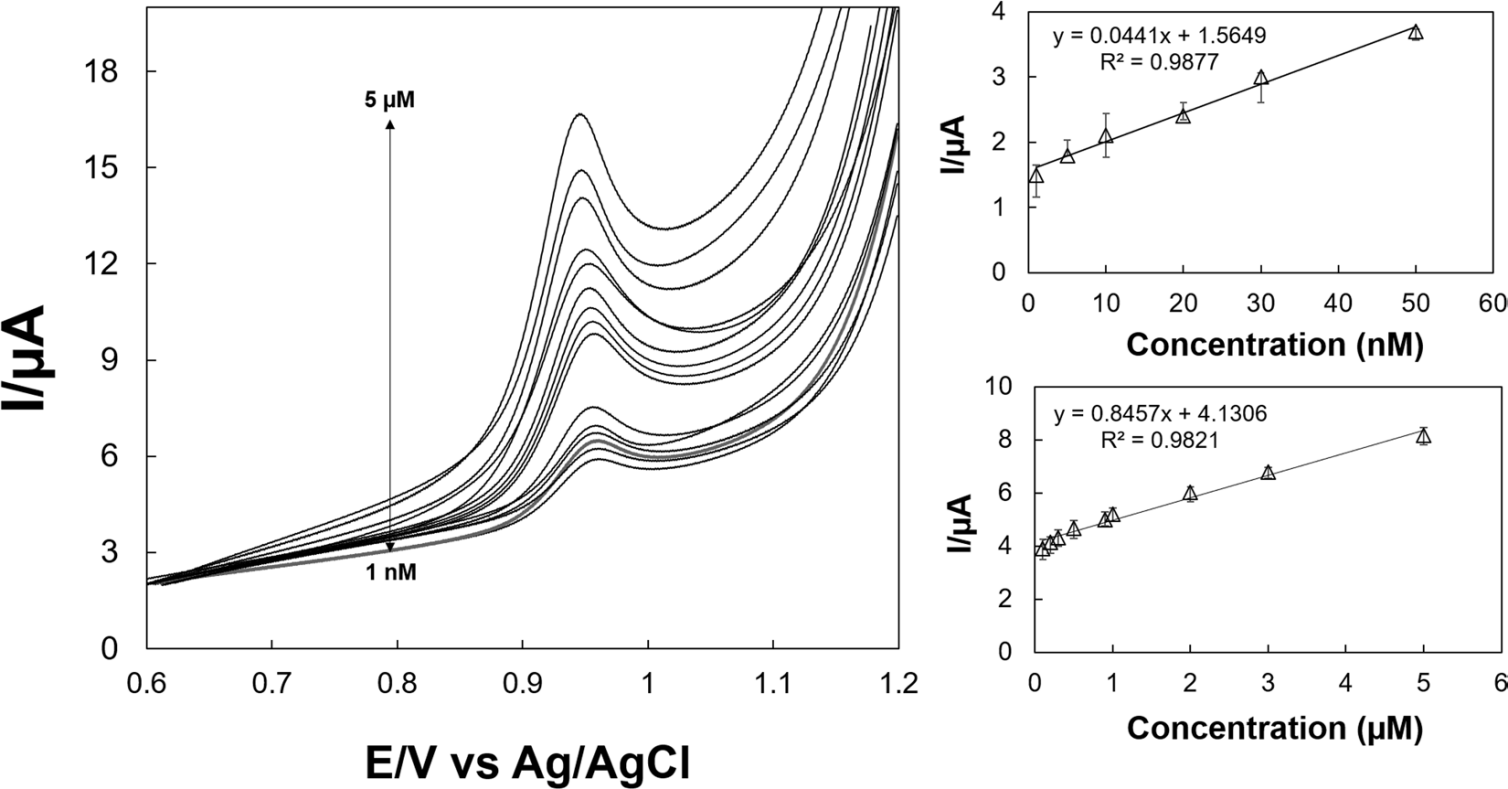
DPVs obtained for determination of CEF using Au-PDA@SiO_2_/rGO/GCE modified electrode in pH 7.0 phosphate buffer at a scan rate of 50 mV s^−1^ with a wide range of concentrations from 1.0 × 10^−9^ M − 5.0 × 10^−8^ and 1.0 × 10^−7^ M − 5.0 × 10^−6^ M. (**a**) All the graphs on the right side show the corresponding calibration curves for low (**b**) and high concentrations (**c**).

Differential pulse voltammograms (DPV) method was used to study the electrochemical behavior of the modified electrode in the presence of different CEF concentration and presented in Fig. 4. An accumulation time of 3 min in 0.1 M PBS (pH 7.0) was used during all experiments. It can be clearly noticed that the oxidative peak current (*I*_*pa*_) increases with the concentration (*C*) of CEF as shown in DPV investigation (Fig. 4a). Two linear segments was observed from the calibration curve of CEF in the range from 1.0 × 10^−9^ to 5.0 × 10^−8^ M (*R*^2^ = 0.9877), and 1.0 × 10^−7^ to 5.0 × 10^−6^ M (*R*^2^ = 0.9821), with a LOD of (S/N = 3) of 1.0 × 10^−10^ M **(**Fig. 4b,c). Table 1 represents the comparison of analytical findings of this study with several modified electrodes reported in literature.

**Table 1:** Comparison of the proposed method with other electrochemical methods recently reported for the determination CEF.

Electrode	Method	Linear range (µM)	LOD (µM)	Reference
AuNPs/Parg/CPE	Linear sweep voltammetry	0.01–100	0.002	^30^
Nano-Pd@ITO	Amperometry analysis	0.1–0.7	0.06	^31^
FeNP/GCE	Differential pulse voltammetry	0.15–25	0.1	^32^
MIP/GNWs@IL-PPNPs/COOH-rGO/GCE	Differential pulse voltammetry	0.003–8.9	0.0001	^1^
NaMM/ERGO/CPE	Differential pulse voltammetry	0.0005–2.4	0.0001	^2^
Au–PtNPs/MWCNT/GCE	Linear sweep voltammetry	0.004–10.0	0.001	^33^
Au-PDA@SiO_2_/rGO/GCE	Differential pulse voltammetry	0.001–5	0.0001	This work

### Reproducibility, stability and selectivity study

To study the long-term stability and reproducibility of the proposed modified electrode, four freshly prepared Au-PDA@SiO_2_/rGO/GCE electrodes were kept in refrigerator for 4 weeks at 4 °C temperature and the voltammograms were measured in PBS solution containing 1.0 × 10^−7^ M CEF. Four replicate measurements show a standard deviation less than ±0.86 for DPV currents which confirms the reproductivity and stability of the proposed sensor. To study the selectivity of the proposed sensor, the effects of some common interferences on the determination of 1 μM CEF was tested. It was observed that 200-fold ascorbic acid, uric acid, and 100-fold glucose, L-glutamic acid did not influence the detection signal (<5%) of CEF measurement. These results indicate the good selectivity of the proposed method.

### Analysis of real samples

In order to investigate the applicability of the proposed electrochemical sensor, recovery tests were performed for CEF in real urine samples. Prior to spiking different concentrations of CEF, healthy human urine samples were diluted 100 times using 0.1 M PBS (pH 7.0). The recovery results obtained from Table 2 demonstrates the possibility of the proposed sensor for real biological samples analysis.

**Table 2:** Determination of spiked CEF in real urine samples.

Sample	Measurement	Added (M)	Detected (M)	Recovery (%)	RSD (%)
Urine 1	1	1.0 × 10^−8^	1.01 × 10^−8^	100.9	2.04
	2	5.0 × 10^−8^	4.87 × 10^−8^	97.40	1.80
	3	1.0 × 10^−6^	0.98 × 10^−6^	98.10	1.23
Urine 2	1	1.0 × 10^−8^	0.97 × 10^−8^	97.21	1.92
	2	5.0 × 10^−8^	5.11 × 10^−8^	102.2	2.15
	3	1.0 × 10^−6^	0.96 × 10^−6^	96.40	1.02

## Conclusion

The present work introduced an innovative Au-PDA@SiO_2_/rGO/GCE modified electrode for the sensitive detection of CEF. The proposed sensor can measure target analytes at very low concentration with a detection limit of 1.0 × 10^−10^ M. Comparing with other CEF sensors, the modified electrode shows wide linear range, long-term stability. Hence, this electrochemical sensor can be used successfully for the determination of CEF in pharmaceutical preparations.
